# Artificial intelligence for predicting treatment responses in autoimmune rheumatic diseases: advancements, challenges, and future perspectives

**DOI:** 10.3389/fimmu.2024.1477130

**Published:** 2024-10-22

**Authors:** Yanli Yang, Yang Liu, Yu Chen, Di Luo, Ke Xu, Liyun Zhang

**Affiliations:** ^1^ Third Hospital of Shanxi Medical University, Shanxi Bethune Hospital, Shanxi Academy of Medical Sciences, Tongji Shanxi Hospital, Taiyuan, China; ^2^ Department of Emergency Medicine, Xinzhou People’s Hospital, Xinzhou, China; ^3^ Department of Health Management, Guangdong Second Provincial General Hospital, Guangzhou, China

**Keywords:** artificial intelligence, machine learning, autoimmune rheumatic diseases, therapeutic response, deep learning

## Abstract

Autoimmune rheumatic diseases (ARD) present a significant global health challenge characterized by a rising prevalence. These highly heterogeneous diseases involve complex pathophysiological mechanisms, leading to variable treatment efficacies across individuals. This variability underscores the need for personalized and precise treatment strategies. Traditionally, clinical practices have depended on empirical treatment selection, which often results in delays in effective disease management and can cause irreversible damage to multiple organs. Such delays significantly affect patient quality of life and prognosis. Artificial intelligence (AI) has recently emerged as a transformative tool in rheumatology, offering new insights and methodologies. Current research explores AI’s capabilities in diagnosing diseases, stratifying risks, assessing prognoses, and predicting treatment responses in ARD. These developments in AI offer the potential for more precise and targeted treatment strategies, fostering optimism for enhanced patient outcomes. This paper critically reviews the latest AI advancements for predicting treatment responses in ARD, highlights the current state of the art, identifies ongoing challenges, and proposes directions for future research. By capitalizing on AI’s capabilities, researchers and clinicians are poised to develop more personalized and effective interventions, improving care and outcomes for patients with ARD.

## Introduction

Autoimmune rheumatic diseases (ARDs) account for a substantial portion of the global disease burden. These distinct disorders arise from abnormal immune responses against normal tissues, attributed to a dysregulated immune system. The reported prevalence of ARDs varies according to studies, ranging from 4.5% to 9.4% (1, 2). Common ARDs include systemic lupus erythematosus (SLE), rheumatoid arthritis (RA), Sjögren’s syndrome (SS), inflammatory myopathies (IM), and systemic sclerosis (SSc). Due to the high heterogeneity among patients, therapeutic outcomes differ significantly, especially with complex treatments such as biological DMARDs (bDMARDs). The challenge of precision medicine in the clinic necessitates ongoing research into accurately predicting treatment responses (3). The rise in big data and advanced analytical techniques has ushered in new possibilities in rheumatology. In recent years, significant innovations have been seen in the digitization of rheumatology. The World Health Organization defines “e-health” as the “cost-effective and secure use of information and communications technologies in support of health and health-related fields, including healthcare services, health surveillance, health literature, and health education, knowledge, and research.” (4). This digital revolution encompasses electronic health records, telemedicine, virtual visits, wearable technology, and mobile health, all enhanced by advancements in information technology and artificial intelligence (AI).

AI originated in 1956 at a workshop at Dartmouth College (5). It is defined as “a system’s ability to interpret external data correctly, to learn from such data, and to use those learnings to achieve specific goals and tasks through flexible adaptation” (6). It often relies on developing sophisticated algorithms based on vast amounts of information to perform independent tasks without human guidance (7, 8). AI is a broad term that encompasses various learning methods including search algorithms, knowledge graphs, natural language processing (NLP), expert systems, evolution algorithms, text and speech synthesis, computer vision, robotics, machine learning (ML), and deep learning (DL) (9). Recently, the terms AI, ML, and DL have been frequently mentioned in both academia and industry and are sometimes used interchangeably due to their overlapping scopes. Generally, AI is the broadest concept, with ML being one of its most important subfields (10). ML focuses on developing models through advanced statistical learning from high-dimensional data without the need for explicit parameter programming (11). DL, a subset of ML, uses neural networks with multiple layers to analyze complex patterns in data.

ML methods can be categorized based on their learning approaches and objectives: supervised learning, unsupervised learning (or clustering), dimensionality reduction, semi-supervised learning, reinforcement learning, and DL (10). ML models can be broadly classified into classical and modern models. Typical semi-supervised learning methods include K-nearest neighbors, logistic regression, decision trees, support vector machines (SVM), and artificial neural networks (10). For unsupervised learning, K-means and principal component analysis are two popular techniques. DL is one of the fastest-growing AI subfields, demonstrating significant potential in solving complex real-world problems. DL architectures have the advantage of solving problems in an end-to-end manner and can be categorized into (1) standard feed-forward neural network (FFNN), recurrent neural network (RNN), convolutional neural network (CNN), and hybrid architectures that combine these basic types (e.g., Siamese networks and transformers). NLP leverages complex ML methods to extract semantic information from text (12). It enables the conversion of unstructured clinical text, such as electronic health records (EHR) data, into structured information that AI algorithms can further process (13).

An explosive growth of AI applications has been seen in medicine. AI in medicine aims to use computer algorithms to process medical data and provide valuable insight to facilitate clinical decision-making (14), such as diagnosis, risk predictions, disease stratification, and treatment selection, ultimately improving health outcomes and enhancing patient experiences. One of the most widely studied AI applications is the simulation of physicians by giving fast and accurate diagnoses (15–18). Several AI diagnosis technologies in auto-diagnosis have already received FDA approval. The first “autonomous” AI diagnostic system to receive FDA approval for the market is the IDx-DR, installed at the University of Iowa to screen patients for diabetes (3). This is the first fully automatic device that can provide screening results without any manual assessment and interpretation from clinicians. In addition to giving a diagnosis alone, AI can be used with human beings to reduce the chance of medical errors and enhance work efficiency (19, 20). Studies have demonstrated a “synergistic effect” when clinicians and AI “collaborate,” resulting in better outcomes than either working alone (21, 22).

As we integrate AI into the era of precision medicine, advanced AI techniques emerge as pivotal solutions for achieving higher prediction performance in ARD research. These techniques model complex associations between patient characteristics and treatment responses, offering real-time insights into disease progression and facilitating swift clinical decision-making for optimal treatment outcomes. For instance, the rapid identification and timely delivery of salvage or alternative therapies can control disease progression and significantly enhance a patient’s overall health. Additionally, AI contributes to a deeper understanding of ARDs origins and progression, enabling more personalized management approaches for ARDs patients.

Despite the advancements in AI, significant challenges persist in the effective and reliable prediction of treatment responses for ARDs patients. The polygenic nature of some ARDs responses necessitates large datasets to identify statistically meaningful associations for biomarker development. Another challenge involves quantifying treatment responses, where achieving consensus can be difficult for some diseases. This discrepancy complicates meaningful comparisons across studies and hampers seamless clinical translation. For example, the target-to-treatment (T2T) approach is prevalent in managing rheumatoid arthritis (RA). Additionally, the prediction timeframe varies across studies; while some predict outcomes months or years after therapy initiation, others focus on predictions before treatment commencement. This review article aims to summarize the current advancements in AI research for predicting treatment responses in ARDs, highlighting the size of study populations as well as the definitions and time frames of treatment response. It concludes by discussing the challenges and future perspectives in this field.

## Literature search

**Figure 1:**
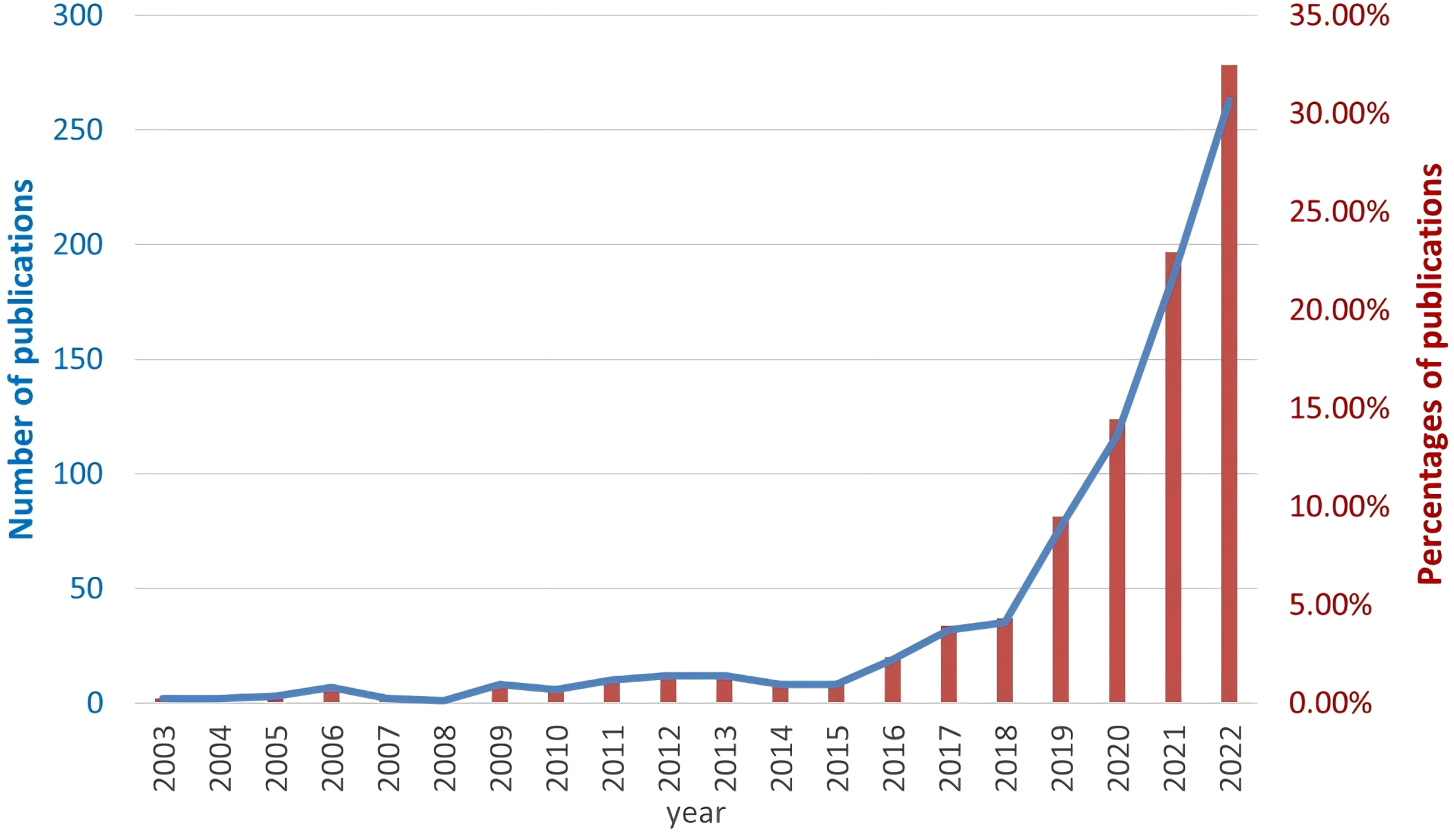
The number of searched publications related to Artificial Intelligence and autoimmune rheumatic diseases by year. The blue line represents the absolute number of researched publications (left y axis) and the red bars represent the percentage of publications (right y axis).

We performed a comprehensive literature search between 2003 and 2022 at the Web of Science Core Collection (WoSCC) database on July 15th, 2023. The WoSCC database was chosen due to its rigorous selection criteria that prioritize high-quality and impactful research. The search query was set as [TS=(“Auto-immune rheumatic diseases” OR “auto-immune diseases” OR “rheumatology” OR “Rheumatic Diseases” OR “Systemic Sclerosis” OR “systemic lupus erythematosus” OR “rheumatoid arthritis” OR “Sjogren’s syndrome” OR “Ankylosing spondylitis” OR “vasculitis” OR “inflammatory myopathies”) AND (“artificial intelligence” OR “machine intelligence” OR “computational intelligence” OR “machine learning” OR “deep learning” OR “neural network” OR “convolutional network” OR “Bayesian*” OR “random forest” OR “reinforcement learning” OR “hierarchical learning” OR “computer vision”)]. The literature types as set as “Articles” and “Reviews” with the exclusion of “Early Access,” “Book Chapters,” “Meeting Abstracts,” “Letters”, etc. A total of 810 articles and reviews were found with a rapid increase of publication number since 2016, as shown in **Figure 1**. Among those, 155 publications focused on RA, 100 on SLE, 44 on ankylosing spondylitis (AS), 32 on SSc, 25 on SS, 19 on osteoarthritis (OA), 13 on dermatomyositis (DM), 10 on juvenile idiopathic arthritis (JIA), 9 on psoriatic arthritis (PSA), 8 on fibromyalgia (FM), 8 on Behçet’s disease (BD), and 8 on vasculitis (VAS) (**Table 1**). The literature discussed six major types of AI applications in ARD, including screening, risk prediction, diagnosis, subtyping, prognosis and endpoint prediction, and monitoring and management. **Table 2** summarizes the aims of selected publications on the applications of AI in predicting the treatment response of ARD patients.

**Table 1:** The number of publications counted by specific autoimmune rheumatic diseases.

Rank	Disease	Counts
1	Rheumatoid arthritis (RA)	155 (19.14%)
2	Systemic lupus erythematosus (SLE)	100 (12.35%)
3	Ankylosing spondylitis (AS)	44 (5.43%)
4	Systemic sclerosis (SSc)	32 (3.95%)
5	Sjögren’s syndrome (SS)	25 (3.09%)
6	Osteoarthritis (OA)	19 (2.35%)
7	Dermatomyositis (DM)	13 (1.60%)
8	Juvenile idiopathic arthritis (JIA)	10 (1.23%)
9	Psoriatic arthritis (PsA)	9 (1.11%)
10	Fibromyalgia (FM)	8 (0.99%)
11	Behçet’s disease (BD)	8 (0.99%)
12	Vasculitis (VAS)	7 (0.86%)

**Table 2:** Summary of selected publications on the applications of AI in predicting treatment response of ARD patients.

Disease	First author	Year of Publishing	Journal	No. of Patients	Aim
RA	Weiyang Tao (42)	2021	Arthritis & Rheumatology	80	Predict Clinical response to adalimumab and etanercept therapy in patients with rheumatoid arthritis
RA	Helen R. Gosselt (26)	2021	J. Pers. Med.	355	Machine learning algorithms and multivariate logistic regression in the prediction of under-response to methotrexate in patients with rheumatoid arthritis
RA	Yuanfang Guan (24)	2019	Arthritis Rheumatol.	2706	Predicting anti-TNF drug responses of rheumatoid arthritis patients by integrating clinical and genetic markers
RA	Darren Plant (44)	2019	Rheumatology (Oxford)	85	Gene expression profiling identifies a classifier of methotrexate non-response in patients with rheumatoid arthritis.
AS	Seulkee Lee (27)	2020	Scientific Reports	595	Predicting early TNF inhibitor users in patients with ankylosing spondylitis
OA	Liangliang Liu (30)	2018	BMC Systems Biology	4796	An interpretable boosting model to predict side effects of analgesics for osteoarthritis
SSc	Showalter K (45).	2021	Ann. Rheum. Dis.	26	To identify molecular signatures that can predict the treatment response (improvers vs. non-improvers)
SSc	Taroni J.N (46).	2017	J. Invest. Dermatol.	Meta-analysis (total 35)	To evaluate gene expressions on skin biopsies and predict response to different treatments
SSc	Ebata S (47).	2022	Rheumatol. Oxf. Engl.	54	To find possible predictors of favorable response to RTX
SSc	Zamanian R.T (48).	2021	Am. J. Respir. Crit. Care Med.	57	To evaluate RTX response in SSc-related PAH
SSc	Franks J.M (49).	2020	Ann. Rheum. Dis.	63	To evaluate stem cell response in severe SSc
IIM inflammatory myopathies	Maria Giovanna Danieli (50)	2022	Autoimmunity Reviews	51	Predicting therapeutic response to intravenous and subcutaneous immunoglobulin in patients with inflammatory myopathies
AOSD	Jinchao Jia (40)	2020	Front. Immunol.	106	Predicting organ involvement and response to glucocorticoids in AOSD patients

## Demographics and statistics

### Risk factor identification and modeling by machine learning

Tumor necrosis factor inhibitors (TNFis) are commonly utilized in treating rheumatoid arthritis (RA), yet their response rate can be as low as 70%. Numerous studies have explored innovative treatment regimens and assessed their efficacy in the RA patient population. For instance, one study developed penalized regression models that utilized clinical and genotypic score covariates (23). These models estimated changes in erythrocyte sedimentation rate (ESR) and swollen joint count (SJC), both of which are components of the Disease Activity Score 28 (DAS28), within three to six months following the initiation of TNFi treatment. However, these models failed to identify strong predictors of TNFi response among the alleles associated with RA development. In another study (24), researchers aimed to predict changes in the disease activity score (ΔDAS28) at 24 months post-baseline assessment. They employed various machine learning techniques—including support vector machine (SVM), Ridge, Random Forest (RF), logistic regression (LR), and Gaussian process regression (GPR), and incorporated demographic, clinical, and genetic characteristics as predictors. Despite the limited contribution of genetic factors to prediction accuracy, the most effective model reached an area under the curve (AUC) value of 0.62 in an independent validation cohort.

Investigations of RA patient response to traditional medications, such as methotrexate (MTX), have attracted researchers’ interest. One study yielded promising performance (AUC = 0.78) in predicting a 6-month post-treatment response (DAS28-CRP) to MTX. The prediction was based on a penalized LR Ridge model trained using the ratio of gene transcript expression values between 4 weeks of treatment and pre-treatment (25). Another recent study compared the performance of ML algorithms, including Lasso, RF, and XGBoost, with LR in predicting under-response to MTX, as measured by DAS28-ESR (26). The authors concluded that the ML algorithm (specifically XGBoost with an AUC of 0.77) has seen minimum advantages over LR (AUC of 0.78) in prediction performance.

Lee et. Al. input clinical and laboratory data from nearly 600 AS patients into an artificial neural network (ANN), as well as other ML algorithms (e.g., XGBoost), to predict early TNF responders (27). The ANN model achieved the best performance (AUC=0.783). In addition, C-reactive protein (CRP) and erythrocyte sediment rate (ESR) were identified as the most important baseline features for predicting early TNFi response using the gradient descent-based feature importance analysis.

Investigators also studied the response of JIA patients to MTX monotherapy and TNFi using the DAS44/ESR-3 index. For the MTX monotherapy, electron medical records before and after drug administration (over three months) were collected from 362 patients and used for response prediction by XGBoost, SVM, LR, and RF modeling (28). Ten pre-treatment predictors and six predictors from a mixture of pre-treatment and post-treatment variables were selected for separate model development by the XGBoost algorithm, achieving a performance of 0.97 and 0.99 for AUC, respectively. Regarding TNFi, the response to the treatment was modeled in 87 patients using the clinical information collected before the administration of the drug (29). Multiple ML algorithms were adopted, including XGBoost, Gradient Enhanced Decision Tree (GBDT), Extreme Random Tree (ET), LR, and RF. The XGBoost model achieved an optimal performance (AUC=0.79) with only four features included as predictors. The XGBoost method was also applied to predict cardiovascular side effects from analgesics for OA treatment and identify high-risk factors (30). More than 300 demographic, anthropometric, comorbidity, hematological, and physical activity characteristics were obtained from 4350 patients provided by the OAI dataset, and 20 high-risk factors were finally identified by the XGBoost predictive model, which achieved an AUC of 0.92.

Gottlieb et al. aimed to predict treatment responses in patients with PsA (n = 2148) (31). In their study, the Bayesian elastic net ML algorithm was used to model the efficacy of the initial dose of the Interleukin (IL)-17A inhibitor, secukinumab. The research analyzed efficacy endpoints—such as ACR20/50, PASI 75/90, PASDAD, and Health Assessment Questionnaire (HAQ)-DI—at week 16, considering 275 predictors. Although no single predictor demonstrated strong discriminatory power, common covariates across all endpoints included baseline inflammation. The study also identified subgroups of patients who might benefit more from the 300 mg dose, notably those not concurrently treated with methotrexate (MTX) or those with psoriasis. The area under the curve (AUC) scores for these endpoints ranged from 0.75 to 0.81.

In a separate study on idiopathic inflammatory myopathy (IIM) (32), AI was utilized to evaluate the response of IIM patients to IVIg and 20% Subcutaneous Immunoglobulin (SCIg) therapy. The diagnosis followed the established EULAR/ACR criteria, with treatment efficacy assessed using parameters such as serum creatine kinase levels, muscle strength, disease activity, and disability. Key predictors for IVIg and 20% SCIg treatments were identified using a combination of supervised machine learning algorithms, including Lasso, Ridge, Elastic Nets, Classification and Regression Trees, and RF. The findings highlighted that muscle strength, as determined by the Manual Muscle Test 8 (MMT8) score during follow-up, was influenced by the presence of dysphagia and skin disease at the start of treatment, as well as the Myositis Intention to Treat Activity Index (MITAX). The correlation between muscle strength and MITAX suggested that IVIg treatment might be more effective in patients with more active systemic disease. Moreover, methods like Elastic Net emerged as the most feasible, efficient, and effective ML approaches for predicting clinical outcomes related to MMT8 and MITAX in myositis.

### Large-scale clinical research by NLP

NLP techniques were also widely applied in ARD research due to their high efficiency in extracting essential information from text-based clinical notes. One study by Cai et al. adopted NLP to identify arthralgia in the clinical records of patients with inflammatory bowel disease (IBW), allowing them to further compare the risk of arthralgia between two treatments, vedolizumab and TNFi. The results showed no significant increase in arthralgia rate with vedolizumab administration (33). This study also demonstrated the superior performance of NLP in arthralgia identification compared to the conventional ICD9 code.

NLP has also been applied with web crawling techniques to extract meaningful information from social media networks. Treato, a once popular data analytics service that combines NLP processing pipelines, medical ontology mapping, classifiers, and sentiment analysis, was employed to extract drug safety data from social media. In one study, Treato analyzed over 785,000 posts related to inflammatory arthritis and investigated patient-reported incidents of herpes zoster associated with arthritis medications, achieving a high positive predictive value of 91% (34). Another study by Dzubur et al. utilized web crawling for social media data extraction. Both Treato and latent Dirichlet allocation (LDA) were adopted for topic modeling to examine the knowledge, attitudes, and beliefs of AS patients about biological therapies (35). They examined 27,000 posts across over 600 social media sites and identified 112 themes, with 36 related explicitly to AS biologic therapies, covering aspects like side effects, biological attributes, and concerns about treatment agents. Treato was also used to analyze RA patients’ perceptions of 13 disease-modifying antirheumatic drugs (DMARDs) (36). For sentiment analysis, NLP helped identify medical concepts and extract patients’ self-descriptions of health conditions and medication experiences. Notably, patients showed more positive attitudes toward biologic DMARDs (bDMARDs) and targeted synthetic DMARDs (tsDMARDs) compared to conventional synthetic DMARDs (csDMARDs). Efficacy and side effects emerged as the most frequently discussed topics.

Other studies have utilized text-mining techniques to identify potential risk factors. For instance, researchers in Japan employed text-mining methods to analyze data from a post-marketing adverse event (AE) reporting database. Their goal was to identify signs and symptoms that appeared before developing severe infections in patients with RA who were treated with the Interleukin (IL)-6 inhibitor tocilizumab (37). Signs and symptoms recorded within 28 days before a severe infection were extracted from unstructured clinical narratives. These were then coded according to the preferred terminology of the Medical Dictionary for Regulatory Activities (MedDRA) and reviewed to assess their commonality in textbooks or clinical practice. The analysis revealed that over 60% of the patients diagnosed with a severe infection had developed indicative signs or symptoms within the 28 days preceding their diagnosis.

### Real-world application in smartphone

AI algorithms have been implemented in smartphone applications to address chronic pain conditions. Smartphone application MyBehaviorCBP leveraged reinforcement learning and sequential decision-making algorithms to analyze routine behaviors and recommend physical activity in patients with chronic back pain (CBP) (38). A similar smartphone-based application driven by multilayer perceptron (MLP) was also introduced to improve self-management of chronic neck and back pain (32).

## Molecular biomarkers

Liu et al. (39) developed a predictive model for TNF blocker treatment response by assessing quantitative changes in IgG galactosylation, alone or in combination with AS-related single nucleotide polymorphisms (SNPs). They created eight ML models, achieving the best AUC of 0.87 for SVM and 0.82 for flexible discriminant analysis (FDA). Meanwhile, glucocorticoids (GCs) are commonly used as first-line therapeutic agents for patients with Adult-Onset Still’s Disease (AOSD). A Chinese research group investigated GC therapeutic response using SVM prediction modeling by considering clinical and laboratory characteristics, including four neutrophil extracellular capture proteins (40). The first SVM model explored whether these proteins could serve as biomarkers for AOSD diagnosis, while the second aimed to predict patient responsiveness or resistance to low-dose GC based on circulating protein levels. The AUC values for the first and second models were 0.88 and 0.91, respectively. In addition, they emphasized the importance of considering the side effects while maximizing the efficacy of the treatment.

Protein kinases have become one of the most critical targets for RA therapy. Protein kinase inhibitors can block the signaling of inflammatory cells by inhibiting kinases, thus inhibiting the transcription of inflammation-related factors. In 2020, Xing et al. established a classification model targeting three kinases (SYK, JAK, and BTK) by combining ML (XGBoost, SVM) models and deep neural networks (41). Morgan fingerprint, Mol2vec descriptor, and MOE descriptor were also used in this study to describe the molecules comprehensively. The model achieved a satisfactory performance with an extensive set of evaluation metrics, including accuracy (0.89 - 0.91), precision (0.90 - 0.93), recall (0.9329 - 0.95), f1 score (0.92 - 0.94), AUC (0.95 - 0.96), Matthew’s correlation coefficient (MCC) (0.74 ~ 0.79), Kappa (0.74 - 0.78), and Brier score (BS) (0.067 - 0.084) reported in the study. In addition, a high recall (92%) and AUC (0.93) were obtained on the external validation set. This integrated model can be used to screen dual-target inhibitors acting on two different signaling pathways, thus producing synergistic therapeutic effects for RA and laying the foundation for subsequent RA drug discovery.

One year later, Tao et al. conducted a study on the gene expression and DNA methylation profiles of peripheral blood mononuclear cells (PBMCs), monocytes, and CD4+ T cells in 80 patients with RA prior to initiating anti-TNF therapy with adalimumab (ADA) or etanercept (ETN) (42). The researchers initially identified transcriptional and epigenetic features associated with treatment responses by analyzing differential gene expression and DNA methylation. Utilizing these features, they developed a machine-learning model using the Random Forest algorithm to predict responses before treatment commenced. Based on differential genes, the model demonstrated an overall accuracy of 85.9% for ADA and 79% for ETN. Even higher accuracy—84.7% for ADA and 88% for ETN—was achieved by considering differentially methylated positions (DMPs). Follow-up studies confirmed the robust performance of these models. Notably, the study uncovered distinct genetic profiles between responders to the two different TNF inhibitors, suggesting divergent mechanisms of treatment response. The researchers concluded that machine learning models based on molecular features could accurately predict pre-treatment responses to ADA and ETN, potentially enhancing personalized anti-TNF therapy.

## Imaging

In recent years, image-based DL and ML algorithms for assessing treatment response have also seen accelerating developments. For instance, Chandrika et al. developed an architecture for assessing bisphosphonate response in 28 patients with chronic nonbacterial osteitis (CNO) based on 55 image pairs (43). The proposed architecture consists of two parts, followed by an integration method that categorizes the scans as “improved,” “poor,” or “stable.” First, the InceptionV3 network extracts features, embeddings, and representations, which are then used in a linear logistic model to generate probability scores. Second, unsupervised clustering techniques labeled the images, and the SVMs generated the probability scores. Despite the less promising results (i.e., low specificity and accuracy), this study highlights the potential of AI for investigating rare rheumatic and musculoskeletal diseases, even in cases with class imbalance and limited training samples.

## Challenges and future perspectives

AI technologies have shown promising potential for predicting treatment responses in ARDs. Advances in this field could represent a significant leap toward precision medicine. By fostering collaborative efforts, embracing interdisciplinary approaches, and leveraging rapidly evolving AI technologies, we can pioneer personalized, effective, and widely accessible therapeutic strategies for ARDs.

Up until now, support vector machines and random forests are the most used machine learning methods with high performance reported in research related to AI and autoimmune diseases (8). Meanwhile, it is still important to be aware of the advantages and disadvantages of different machine learning models in order to choose the most appropriate ones for the target scenario. For example, decision trees are simple and highly interpretable, but often reported to achieve inferior performance due to its low stability under small sample size. The use of random forest methods can improve performance, but at the cost of losing interpretability. K-nearest neighbor is another nonparametric method that performs well in separating classes with complex boundaries, but such high sensitivity often leads to overfitting, resulting in poor classification results during validations. Therefore, the most suitable model should be evaluated based on a combination of factors including clinical endpoint, dataset characteristics and the need for interpretability.

Meanwhile, challenges still lie ahead, and questions that remain unsolved could shape the future of ARDs research (1). One key challenge is the large clinical and molecular heterogeneities among patients with ARDs, which could significantly impede effective treatment allocations and reduce treatment efficacy (2, 51). The insufficient sample size during analysis, particularly on some rare ARDs such as mixed connective tissue disease, polymyositis, SS, and vasculitis, could prevent researchers from drawing meaningful conclusions. Multi-center research or database sharing could be solutions to the current challenge (3). Compared to standard clinical experiments, the retrospective collected data may suffer from low data quality (e.g., lack of control of confounding factors) and lack of standardization (52). Those disadvantages could significantly impede the research and deployment cycle and increase the risk of false discovery. How to effectively standardize the data without sacrificing the richness of information is also an issue that needs to be addressed (5). There still exists the risk of algorithmic biases that reinforce discriminatory practices based on race, gender, or other characteristics (4, 53) . Finally, safeguarding patient medical information presents challenges as well. A data breach can have catastrophic consequences. Implementing blockchain technology could be one solution to enhance data security, but it may restrict geographical collaborations in digital healthcare.

Large models with complex structures and large number of parameters are preferred in research due their high prediction accuracies and versatilities. However, they can be computationally expensive in the application stage, requiring more advanced hardware and consuming more energy. In future research, model compression techniques, such as weight pruning, knowledge distillation, and quantization, can reduce the size and computational requirements of the model while maintaining its performance. In addition, researchers can explore lightweight neural network architectures such as MobileNets and EfficientNets (54, 55), which are designed for mobile and edge computing environments for efficient inference. These approaches not only help to reduce the energy consumption of devices, but also reduce the carbon footprint of data processing, driving the development of green computing.

## References

[1] Cooper GS Bynum ML Somers EC. Recent insights in the epidemiology of autoimmune diseases: improved prevalence estimates and understanding of clustering of diseases. J Autoimmun. (2009) 33:197–207. doi: 10.1016/j.jaut.2009.09.008 19819109 PMC2783422

[2] Hayter SM Cook MC. Updated assessment of the prevalence, spectrum and case definition of autoimmune disease. Autoimmun Rev. (2012) 11:754–65. doi: 10.1016/j.autrev.2012.02.001 22387972

[3] He J Baxter SL Xu J Xu J Zhou X Zhang K. The practical implementation of artificial intelligence technologies in medicine. Nat Med. (2019) 25:30–6. doi: 10.1038/s41591-018-0307-0 PMC6995276 30617336

[4] Conrad K Shoenfeld Y Fritzler MJ. Precision health: A pragmatic approach to understanding and addressing key factors in autoimmune diseases. Autoimmun Rev. (2020) 19:102508. doi: 10.1016/j.autrev.2020.102508 32173518

[5] McCarthy J Minsky ML Rochester N Shannon CE. A Proposal for the Dartmouth Summer Research Project on Artificial Intelligence, August 31, 1955 Vol. 27. AI Magazine (2006). p. 12. doi: 10.1609/aimag.v27i4.1904

[6] Haenlein M Kaplan A. A brief history of artificial intelligence: on the past, present, and future of artificial intelligence. California Manage Review. (2019) 61:5–14. doi: 10.1177/0008125619864925

[7] LeCun Y Bengio Y Hinton G. Deep learning. Nature. (2015) 521:436–44. doi: 10.1038/nature14539 26017442

[8] Stafford IS Kellermann M Mossotto E Beattie RM MacArthur BD Ennis S. A systematic review of the applications of artificial intelligence and machine learning in autoimmune diseases. NPJ Digit Med. (2020) 3:30. doi: 10.1038/s41746-020-0229-3 32195365 PMC7062883

[9] Xu Y Liu X Cao X Huang C Liu E Qian S. Artificial intelligence: A powerful paradigm for scientific research. Innovation. (2021) 2:100179. doi: 10.1016/j.xinn.2021.100179 34877560 PMC8633405

[10] Mukhamediev RI Popova Y Kuchin Y Zaitseva E Kalimoldayev A Symagulov A. Review of artificial intelligence and machine learning technologies: classification, restrictions, opportunities and challenges. Mathematics. (2022) 10:2552. doi: 10.3390/math10152552

[11] Valiant LG. A theory of the learnable. Commun ACM. (1984) 27:1134–42. doi: 10.1145/1968.1972

[12] Turner CA Jacobs AD Marques CK Oates JC Kamen DL Anderson PE. Word2Vec inversion and traditional text classifiers for phenotyping lupus. BMC Med Inform Decis. (2017) 17:126. doi: 10.1186/s12911-017-0518-1 PMC5568290 28830409

[13] Shrestha A Mahmood A. Review of deep learning algorithms and architectures. IEEE Access. (2019) 7:53040–65. doi: 10.1109/ACCESS.2019.2912200

[14] Murdoch TB Detsky AS. The inevitable application of big data to health care. Jama. (2013) 309:1351–2. doi: 10.1001/jama.2013.393 23549579

[15] Gulshan V Peng L Coram M Stumpe MC Wu D Narayanaswamy A. Development and validation of a deep learning algorithm for detection of diabetic retinopathy in retinal fundus photographs. Jama. (2016) 316:2402–10. doi: 10.1001/jama.2016.17216 27898976

[16] Kermany DS Goldbaum M Cai W Valentim CCS Liang H Baxter SL. Identifying medical diagnoses and treatable diseases by image-based deep learning. Cell. (2018) 172:1122–31.e9. doi: 10.1016/j.cell.2018.02.010 29474911

[17] Esteva A Kuprel B Novoa RA Ko J Swetter SM Blau HM. Dermatologist-level classification of skin cancer with deep neural networks. Nature. (2017) 542:115–8. doi: 10.1038/nature21056 PMC8382232 28117445

[18] Cheng JZ Ni D Chou YH Qin J Tiu CM Chang YC. Computer-aided diagnosis with deep learning architecture: applications to breast lesions in US images and pulmonary nodules in CT scans. Sci Rep. (2016) 6:24454. doi: 10.1038/srep24454 27079888 PMC4832199

[19] Jiang F Jiang Y Zhi H Dong Y Li H Ma S. Artificial intelligence in healthcare: past, present and future. Stroke Vasc Neurol. (2017) 2:230–43. doi: 10.1136/svn-2017-000101 PMC5829945 29507784

[20] Johnson KW Torres Soto J Glicksberg BS Shameer K Miotto R Ali M. Artificial intelligence in cardiology. J Am Coll Cardiol. (2018) 71:2668–79. doi: 10.1016/j.jacc.2018.03.521 29880128

[21] Chen H Wang N Du X Mei K Zhou Y Cai G. Classification prediction of breast cancer based on machine learning. Comput Intell Neurosci. (2023) 2023:6530719. doi: 10.1155/2023/6530719 36688223 PMC9848804

[22] Lakhani P Sundaram B. Deep learning at chest radiography: automated classification of pulmonary tuberculosis by using convolutional neural networks. Radiology. (2017) 284:574–82. doi: 10.1148/radiol.2017162326 28436741

[23] Spiliopoulou A Colombo M Plant D Nair N Cui J Coenen MJ. Association of response to TNF inhibitors in rheumatoid arthritis with quantitative trait loci for CD40 and CD39. Ann Rheum Dis. (2019) 78:1055–61. doi: 10.1136/annrheumdis-2018-214877 PMC6669378 31036624

[24] Guan Y Zhang H Quang D Wang Z Parker SCJ Pappas DA. Machine learning to predict anti-tumor necrosis factor drug responses of rheumatoid arthritis patients by integrating clinical and genetic markers. Arthritis Rheumatol. (2019) 71:1987–96. doi: 10.1002/art.41056 31342661

[25] Plant D Maciejewski M Smith S Nair N Hyrich K Ziemek D. Profiling of gene expression biomarkers as a classifier of methotrexate nonresponse in patients with rheumatoid arthritis. Arthritis Rheumatol. (2019) 71:678–84. doi: 10.1002/art.40810 PMC9328381 30615300

[26] Gosselt HR Verhoeven MMA Bulatović-Ćalasan M Welsing PM de Rotte M Hazes JMW. Complex machine-learning algorithms and multivariable logistic regression on par in the prediction of insufficient clinical response to methotrexate in rheumatoid arthritis. J Pers Med. (2021) 11:14. doi: 10.3390/jpm11010044 PMC7828730 33466633

[27] Lee S Eun Y Kim H Cha HS Koh EM Lee J. Machine learning to predict early TNF inhibitor users in patients with ankylosing spondylitis. Sci Rep. (2020) 10:20299. doi: 10.1038/s41598-020-75352-7 33219239 PMC7679386

[28] Mo X Chen X Li H Li J Zeng F Chen Y. Early and accurate prediction of clinical response to methotrexate treatment in juvenile idiopathic arthritis using machine learning. Front Pharmacol. (2019) 10:1155. doi: 10.3389/fphar.2019.01155 31649533 PMC6791251

[29] Mo X Chen X Ieong C Zhang S Li H Li J. Early prediction of clinical response to etanercept treatment in juvenile idiopathic arthritis using machine learning. Front Pharmacol. (2020) 11:1164. doi: 10.3389/fphar.2020.01164 32848772 PMC7411125

[30] Liu L Yu Y Fei Z Li M Wu FX Li HD. An interpretable boosting model to predict side effects of analgesics for osteoarthritis. BMC Syst Biol. (2018) 12:105. doi: 10.1186/s12918-018-0624-4 30463545 PMC6249730

[31] Gottlieb AB Mease PJ Kirkham B Nash P Balsa AC Combe B. Secukinumab efficacy in psoriatic arthritis: machine learning and meta-analysis of four phase 3 trials. J Clin Rheumatol. (2021) 27:239–47. doi: 10.1097/rhu.0000000000001302 PMC8389345 32015257

[32] Lo WLA Lei D Li L Huang DF Tong KF. The perceived benefits of an artificial intelligence-embedded mobile app implementing evidence-based guidelines for the self-management of chronic neck and back pain: observational study. JMIR Mhealth Uhealth. (2018) 6:e198. doi: 10.2196/mhealth.8127 30478019 PMC6288595

[33] Cai T Lin TC Bond A Huang J Kane-Wanger G Cagan A. The association between arthralgia and vedolizumab using natural language processing. Inflammation Bowel Dis. (2018) 24:2242–6. doi: 10.1093/ibd/izy127 PMC6140445 29846617

[34] Curtis JR Chen L Higginbotham P Nowell WB Gal-Levy R Willig J. Social media for arthritis-related comparative effectiveness and safety research and the impact of direct-to-consumer advertising. Arthritis Res Ther. (2017) 19:48. doi: 10.1186/s13075-017-1251-y 28270190 PMC5341200

[35] Dzubur E Khalil C Almario CV Noah B Minhas D Ishimori M. Patient concerns and perceptions regarding biologic therapies in ankylosing spondylitis: insights from a large-scale survey of social media platforms. Arthritis Care Res (Hoboken). (2019) 71:323–30. doi: 10.1002/acr.23600 PMC6946060 29781587

[36] Sharma C Whittle S Haghighi PD Burstein F Sa’adon R Keen HI. Mining social media data to investigate patient perceptions regarding DMARD pharmacotherapy for rheumatoid arthritis. Ann Rheum Dis. (2020) 79:1432–7. doi: 10.1136/annrheumdis-2020-217333 PMC7569383 32883653

[37] Atsumi T Ando Y Matsuda S Tomizawa S Tanaka R Takagi N. Prodromal signs and symptoms of serious infections with tocilizumab treatment for rheumatoid arthritis: Text mining of the Japanese postmarketing adverse event-reporting database. Mod Rheumatol. (2018) 28:435–43. doi: 10.1080/14397595.2017.1366007 28880689

[38] Rabbi M Aung MS Gay G Reid MC Choudhury T. Feasibility and acceptability of mobile phone-based auto-personalized physical activity recommendations for chronic pain self-management: pilot study on adults. J Med Internet Res. (2018) 20:e10147. doi: 10.2196/10147 30368433 PMC6229514

[39] Liu J Zhu Q Han J Zhang H Li Y Ma Y. IgG Galactosylation status combined with MYOM2-rs2294066 precisely predicts anti-TNF response in ankylosing spondylitis. Mol Med. (2019) 25:25. doi: 10.1186/s10020-019-0093-2 31195969 PMC6567531

[40] Jia J Wang M Ma Y Teng J Shi H Liu H. Circulating neutrophil extracellular traps signature for identifying organ involvement and response to glucocorticoid in adult-onset still’s disease: A machine learning study. Front Immunol. (2020) 11:563335. doi: 10.3389/fimmu.2020.563335 33240258 PMC7680913

[41] Xing G Liang L Deng C Hua Y Chen X Yang Y. Activity prediction of small molecule inhibitors for antirheumatoid arthritis targets based on artificial intelligence. ACS Comb Sci. (2020) 22:873–86. doi: 10.1021/acscombsci.0c00169 33146518

[42] Tao W Concepcion AN Vianen M Marijnissen ACA Lafeber F Radstake T. Multiomics and machine learning accurately predict clinical response to adalimumab and etanercept therapy in patients with rheumatoid arthritis. Arthritis Rheumatol. (2021) 73:212–22. doi: 10.1002/art.41516 PMC7898388 32909363

[43] Bhat CS Chopra M Andronikou S Paul S Wener-Fligner Z Merkoulovitch A. Artificial intelligence for interpretation of segments of whole body MRI in CNO: pilot study comparing radiologists versus machine learning algorithm. Pediatr Rheumatol Online J. (2020) 18:47. doi: 10.1186/s12969-020-00442-9 32517764 PMC7285749

[44] Bansard C Lequerré T Derambure C Vittecoq O Hiron M Daragon A. Gene profiling predicts rheumatoid arthritis responsiveness to IL-1Ra (anakinra). Rheumatol (Oxford). (2011) 50:283–92. doi: 10.1093/rheumatology/keq344 21059672

[45] Showalter K Spiera R Magro C Agius P Martyanov V Franks JM. Machine learning integration of scleroderma histology and gene expression identifies fibroblast polarisation as a hallmark of clinical severity and improvement. Ann Rheum Dis. (2021) 80:228–37. doi: 10.1136/annrheumdis-2020-217840 PMC8600653 33028580

[46] Taroni JN Martyanov V Mahoney JM Whitfield ML. A functional genomic meta-analysis of clinical trials in systemic sclerosis: toward precision medicine and combination therapy. J Invest Dermatol. (2017) 137:1033–41. doi: 10.1016/j.jid.2016.12.007 PMC8190797 28011145

[47] Ebata S Oba K Kashiwabara K Ueda K Uemura Y Watadani T. Predictors of rituximab effect on modified Rodnan skin score in systemic sclerosis: a machine-learning analysis of the DesiReS trial. Rheumatol (Oxford). (2022) 61:4364–73. doi: 10.1093/rheumatology/keac023 35136981

[48] Zamanian RT Badesch D Chung L Domsic RT Medsger T Pinckney A. Safety and efficacy of B-cell depletion with rituximab for the treatment of systemic sclerosis-associated pulmonary arterial hypertension: A multicenter, double-blind, randomized, placebo-controlled trial. Am J Respir Crit Care Med. (2021) 204:209–21. doi: 10.1164/rccm.202009-3481OC PMC8650794 33651671

[49] Franks JM Martyanov V Wang Y Wood TA Pinckney A Crofford LJ. Machine learning predicts stem cell transplant response in severe scleroderma. Ann Rheum Dis. (2020) 79:1608–15. doi: 10.1136/annrheumdis-2020-217033 PMC8582621 32933919

[50] Danieli MG Tonacci A Paladini A Longhi E Moroncini G Allegra A. A machine learning analysis to predict the response to intravenous and subcutaneous immunoglobulin in inflammatory myopathies. A proposal for a future multi-omics approach in autoimmune diseases. Autoimmun Rev. (2022) 21:103105. doi: 10.1016/j.autrev.2022.103105 35452850

[51] Kaplan MJ. Navigating an enigma: the continuing journey of autoimmunity discoveries. J Clin Invest. (2024) 134:e182287. doi: 10.1172/JCI182287 38828730 PMC11142729

[52] Kruse CS Goswamy R Raval Y Marawi S. Challenges and opportunities of big data in health care: A systematic review. JMIR Med Inform. (2016) 4:e38. doi: 10.2196/medinform.5359 27872036 PMC5138448

[53] Char DS Shah NH Magnus D. Implementing machine learning in health care - addressing ethical challenges. N Engl J Med. (2018) 378:981–3. doi: 10.1056/NEJMp1714229 PMC5962261 29539284

[54] Tan M Le QV. EfficientNet: rethinking model scaling for convolutional neural networks. (2019). doi: 10.48550/arXiv.1905.11946

[55] Howard AG Zhu M Chen B Kalenichenko D Wang W Weyand T. MobileNets: efficient convolutional neural networks for mobile vision applications. (2017). doi: 10.48550/arXiv.1704.04861

